# The MATE trial: a multicentre, mixed-methodology, pilot, randomised controlled trial in neovascular age-related macular degeneration

**DOI:** 10.1186/s40814-023-01288-0

**Published:** 2023-04-20

**Authors:** Archana Airody, Heidi A. Baseler, Julie Seymour, Victoria Allgar, Rajarshi Mukherjee, Louise Downey, Sushma Dhar-Munshi, Sajjad Mahmood, Konstantinos Balaskas, Theo Empeslidis, Rachel L. W. Hanson, Tracey Dorey, Tom Szczerbicki, Sobha Sivaprasad, Richard P. Gale

**Affiliations:** 1 Academic Unit of Ophthalmology, York & Scarborough Teaching Hospitals NHS Foundation Trust, York, YO31 8HE UK; 2 Department of Psychology, University of York, York, UK; 3 Hull York Medical School, University of York, York, UK; 4 Hull York Medical School, University of Hull, Hull, UK; 5 Peninsula Medical School, University of Plymouth, Plymouth, UK; 6 Leeds Teaching Hospital NHS Foundation Trust, Leeds, UK; 7 Hull and East Yorkshire NHS Trust, Hull, UK; 8 Kings Mill Hospital, Sherwood Forest Hospitals NHS Foundation Trust, Sutton-in-Ashfield, UK; 9 University of Manchester, Manchester, UK; 10 NIHR Moorfields Biomedical Research Centre, Moorfields Eye Hospital NHS Foundation Trust, London, UK; 11 Leicester Royal Infirmary, University Hospitals of Leicester NHS Trust, Leicester, UK; 12 Research and Development, York & Scarborough Teaching Hospitals NHS Foundation Trust, York, UK

**Keywords:** Pilot, Randomised controlled trial, Mixed methodology, Neovascular age-related macular degeneration

## Abstract

**Background/objectives:**

In healthcare research investigating complex interventions, gaps in understanding of processes can be filled by using qualitative methods alongside a quantitative approach. The aim of this mixed-methods pilot trial was to provide feasibility evidence comparing two treatment regimens for neovascular age-related macular degeneration (nAMD) to inform a future large-scale randomised controlled trial (RCT).

**Subjects/methods:**

Forty-four treatment-naïve nAMD patients were followed over 24 months and randomised to one of two treatment regimens: standard care (SC) or treat and extend (T&E). The primary objective evaluated feasibility of the MATE trial via evaluations of screening logs for recruitment rates, nonparticipation and screen fails, whilst qualitative in-depth interviews with key study staff evaluated the recruitment phase and running of the trial. The secondary objective assessed changes in visual acuity and central retinal thickness (CRT) between the two treatment arms.

**Results:**

The overall recruitment rate was 3.07 participants per month with a 40.8% non-participation rate, 18.51% screen-failure rate and 15% withdrawal/non-completion rate. Key themes in the recruitment phase included human factors, protocol-related issues, recruitment processes and challenges. Both treatment regimens showed a trend towards a visual acuity gain at month 12 which was not maintained at month 24, whilst CRT reduced similarly in both regimens over the same time period. These were achieved with one less treatment following a T&E regimen.

**Conclusion:**

This mixed-methodology, pilot RCT achieved its pre-defined recruitment, nonparticipation and screen failure rates, thus deeming it a success. With some minor protocol amendments, progression to a large-scale RCT will be achievable.

**Supplementary Information:**

The online version contains supplementary material available at 10.1186/s40814-023-01288-0.

## Key message regarding feasibility

- What uncertainties existed regarding feasibility?
  - ◦ There is a lack of information relating to the screening and recruitment processes, including recruitment time and target, in ophthalmology clinical research.
- What are the key feasibility findings?
  - ◦ Quantitative analysis revealed the overall recruitment rate was 3.07 participants per month with a 40.8% non-participation rate, 18.51% screen-failure rate and 15% withdrawal/non-completion rate.
  - ◦ Qualitative analysis with key trial staff revealed key themes in the recruitment phase included human factors, protocol-related issues, recruitment processes and challenges.
- What are the implications of the feasibility findings for the design of the main study?
  - ◦ Progression of this trial to a large-scale RCT can be achieved with minor amendments to the study protocol, identified via the study findings.

## Introduction

When evaluating healthcare interventions, the most effective and reliable method used is a randomised controlled trial (RCT). Importantly, there are a number of aspects which can impact the success of a RCT if not considered thoroughly beforehand. Such aspects may include assessing recruitment rates and screening logs and practicality of a study amongst others; however, these can be evaluated in a pilot, feasibility trial. According to the Consolidated Standards of Reporting Trials (CONSORT) guidelines [1], randomised controlled trials (RCT) evaluate the efficacy and/or the effectiveness of an intervention. Randomised pilot and feasibility trials on the other hand are conducted in advance of a future RCT with the primary aim to assess the feasibility of conducting a future RCT based on whether the future trial can be done, should be done and, if so, how [1].

Many RCTs experience difficulty recruiting both to target and to time, resulting in underpowered studies, often costly extensions or early study closures [2–4]. Understanding the possible barriers in the recruitment process and identifying ways to overcome them may help to alleviate some of these challenges. Collecting and recording data pertaining to patients screened for a RCT are a current recommendation in CONSORT reporting guidelines [5] and a consideration under Good Clinical Practice (GCP) [6]. This data, in the form of a screening log, should include the numbers assessed for eligibility, those meeting the exclusion and inclusion criteria and those who declined [5]. The SEAR (screening, eligibility, approach, randomisation) framework was developed to encourage the collection of recruitment information, to identify recruitment obstacles and to facilitate improvements in the recruitment process in clinical trials [7].

Evaluations of recruitment rates and screening logs provide quantitative analysis of a study; however, conducting in-depth interviews with key staff involved in the study provides a qualitative assessment of the recruitment phase of a study, the challenges faced and ideas for improvement. Therefore, in healthcare research, there has been an increase in the use of mixed-methodology designs combining quantitative with qualitative data in a study analysis. Research has shown that in complex interventions, gaps in understanding of processes can be filled by using qualitative methods alongside a quantitative approach [8]. In conjunction with RCTs, qualitative methodologies have enhanced our understanding when exploring experiences of trial processes, acceptability, practicality and implementation of a study [8–11].

Age-related macular degeneration (AMD) is one of the leading causes of sight loss in the developed world [12]. The prevalence of the late form of AMD, neovascular-AMD (nAMD), is estimated at 263,000 cases in the UK alone [13]. Whilst the landmark RCTs investigating nAMD, including ANCHor and Marina [14, 15], CATT [16], IVAN [17] and VIEW [18] to name a few, have all expressed the benefits of different treatments and regimens for nAMD, there is a lack of information pertaining to the screening process in general in ophthalmology research. There is also a lack of information regarding recruitment in ophthalmology clinical trials, specifically in AMD and nAMD. Slow recruitment has been noted in the SCORE-CRVO (Standard Care versus Corticosteroid for Retinal Vein Occlusion - Central Retinal Vein Occlusion) trial which led to the SCORE 2 team implementing techniques to enhance recruitment, evaluating their usefulness via questionnaires [19]. The authors reported that recruitment was facilitated by imposing less restrictive eligibility criteria, the ability to screen and randomise on the same day and not including a sham arm [19]. Interestingly, data from the National Institute for Health and Care Research (NIHR) reported that in the first quarter of 2022, just 51.4% of 1.191 research trials conducted within National Health Service (NHS) settings achieved a pre-defined recruitment target and recruited to time. Whilst this figure is not specific to ophthalmology, it does show that in general, challenges remain in achieving recruitment rates in clinical research [20].

The current trial was developed as a multicentre, mixed-methodology, pilot, randomised controlled trial (RCT) to evaluate the feasibility of conducting a large-scale RCT comparing two treatment regimens in neovascular age-related macular degeneration (nAMD). To record the potential challenges and ways to overcome them in a large-scale RCT, the current study employed qualitative and quantitative methods used in parallel to analyse the recruitment phase, set-up and running of the study via assessments of screening logs and face-to-face interviews with trial staff.

## Subjects and methods

The ‘treating neovascular age-related Macular degeneration with Aflibercept: a multi-centre randomised controlled trial comparing standard care with an individualised Treat-and-Extend regimen’ (MATE) trial was a multicentre, pilot, RCT comparing two treatment regimens of aflibercept for neovascular age-related macular degeneration (nAMD). The MATE study was conducted in six NHS medical retina units across the UK from December 2015 to January 2019. Written informed consent was obtained from all participants. Ethical approval was granted by the NHS Research and Ethics Committee (IRAS: 178,790, ISRCTN: 58,955,026; EUDRACT: 2015–002,302-36). This study followed the tenets of the Declaration of Helsinki.

### Subjects

**Fig. 1:**
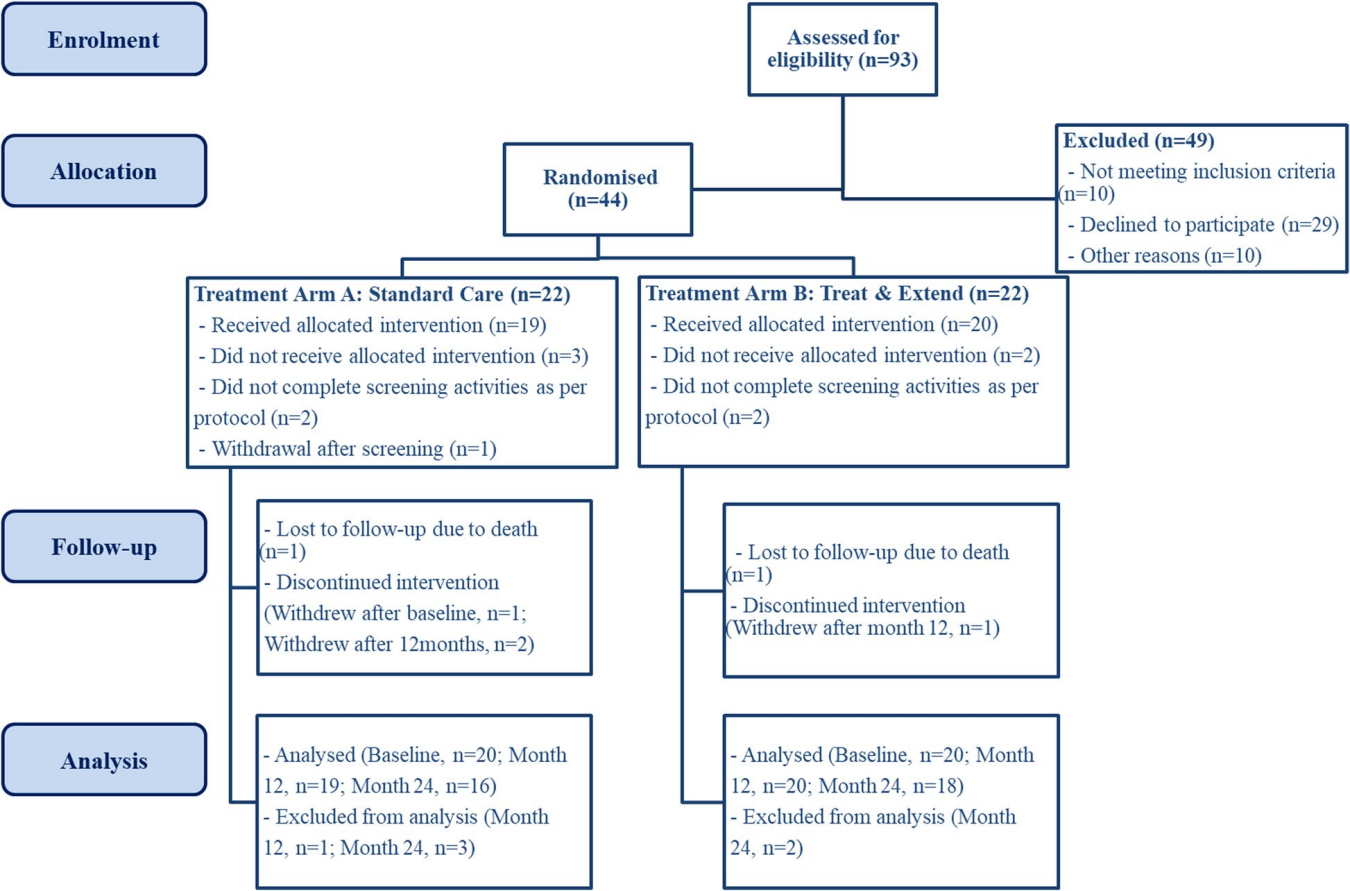
CONSORT-style diagram showing patient flow through the MATE trial

A total of ninety-three participants were approached to take part in this study between December 2015 and January 2017 across all study sites. Whilst no a priori power calculation was completed due to this being a pilot trial, the intended sample size was 40 participants based on an expectation that each study site would recruit 8 participants in accordance with real-world nAMD treatment trials. According to the eligibility criteria, all participants were diagnosed with active, treatment-naive nAMD, had a visual acuity of 78–24 ETDRS letters at screening and baseline in the study eye, aged at least 50 years and able and willing to comply to all study visits at the frequency required. For a full list of study inclusion and exclusion criteria, please see Supplementary Table 1. Following the exclusion of forty-nine participants, the remaining forty-four were randomised into either the standard care (SC) or treat-and-extend (T&E) treatment regimen (Fig. 1). A 1:1 randomisation was performed allocating each participant into one of the treatment regimens. This service was provided by a web-based system, SealedEnvelope.com (https://www.sealedenvelope.com/), and conducted centrally by the trial manager responsible for the whole trial. The trial manager was independent to the study staff. Optometrists performing visual acuity assessments were also masked to the study participants’ allocation following standard practice in nAMD treatment trials. Baseline demographics of the final cohort can be found in Table 1.

**Table 1:** Baseline demographics of participants in the MATE trial

	**Main MATE trial**	
	**Treatment arm A: SC**	**Treatment arm B: T&E**
**Mean age (years; SD)**	78.98 (7.7)	78.4 (6.5)
**Gender**		
Female (%)	11 (55%)	11 (55%)
Male (%)	9 (45%)	9 (45%)
**BCVA (SD)**	60.8 (12.5)	63.7 (10.0)
**CRT (µm; SD)**	414.3 (144.5)	406.6 (114.6)

*SC* standard care, *T&E* treat and extend, *SD* standard deviation, *BCVA* best-corrected visual acuity, *CRT* central retinal thickness

### Methods

**Fig. 2:**
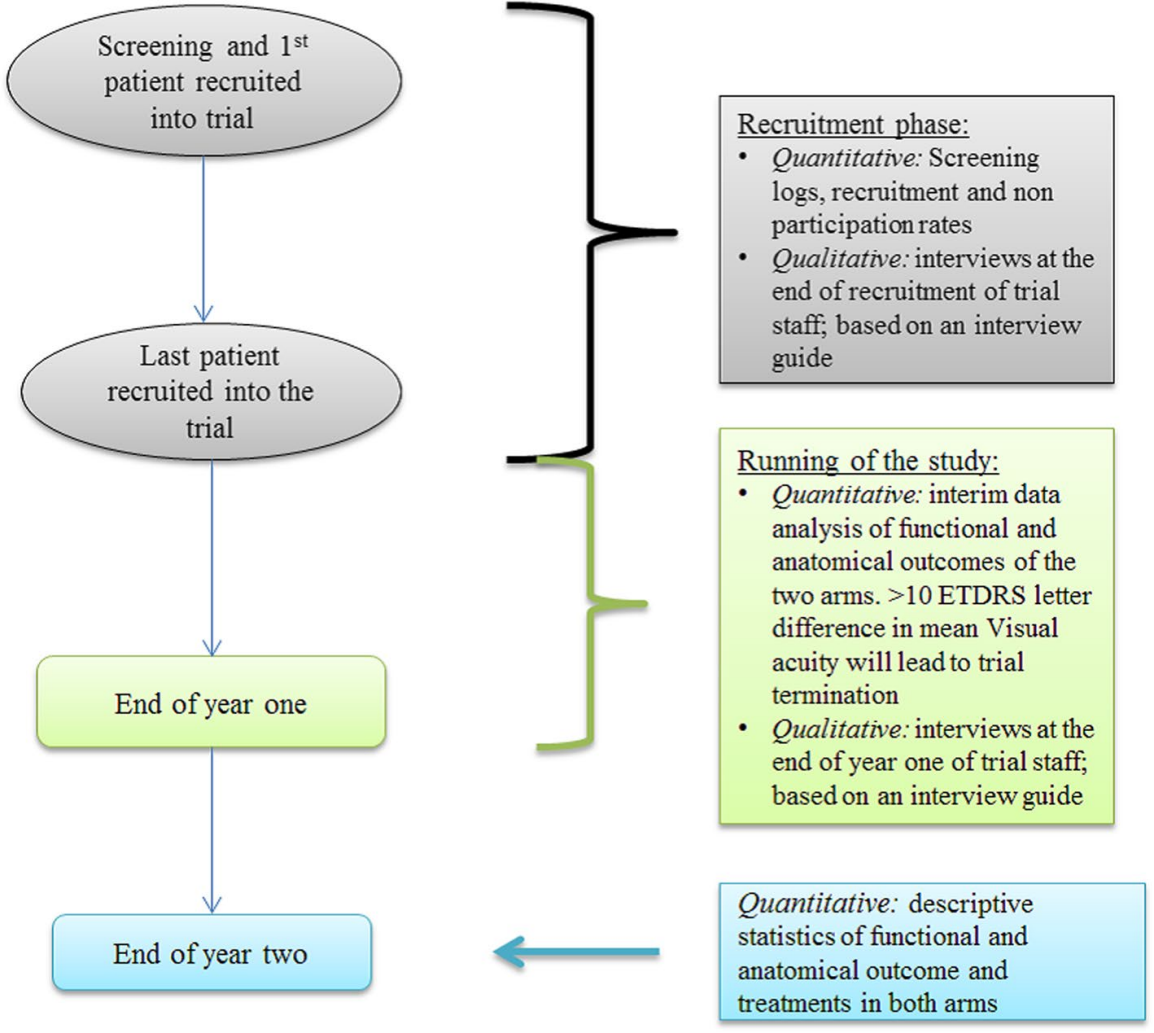
The interplay of the qualitative and quantitative components in the MATE trial

This trial employed a mixed methodology with parallel data analysis (Fig. 2). The primary objective used quantitative and qualitative techniques to evaluate the feasibility and acceptability of the MATE trial in providing adequate evidence to inform a large-scale RCT following the 2010 CONSORT guidelines [1] (Table 2). All participants were identified and recruited by the primary investigator (PI) at each study site following a convenience sampling strategy. Research nurses were involved in facilitating the study under the supervision of the site-specific PI but were not involved in participant identification or recruitment.

**Table 2:** CONSORT 2010 checklist for reporting pilot, randomised controlled trials (RCTs) and the location of information for the MATE trial

Section/topic	Item no	Checklist item	Reported on page no
**Title and abstract**			
	1a	Identification as a pilot or feasibility randomised trial in the title	1
	1b	Structured summary of pilot trial design, methods, results and conclusions (for specific guidance, see CONSORT abstract extension for pilot trials)	3
**Introduction**			
Background and objectives	2a	Scientific background and explanation of rationale for future definitive trial and reasons for randomised pilot trial	4
	2b	Specific objectives or research questions for pilot trial	4
**Methods**			
Trial design	3a	Description of pilot trial design (such as parallel, factorial) including allocation ratio	5
	3b	Important changes to methods after pilot trial commencement (such as eligibility criteria), with reasons	7
Participants	4a	Eligibility criteria for participants	5
	4b	Settings and locations where the data were collected	5
	4c	How participants were identified and consented	5
Interventions	5	The interventions for each group with sufficient details to allow replication, including how and when they were actually administered	5
Outcomes	6a	Completely defined prespecified assessments or measurements to address each pilot trial objective specified in 2b, including how and when they were assessed	5/6
	6b	Any changes to pilot trial assessments or measurements after the pilot trial commenced, with reasons	n/a
	6c	If applicable, prespecified criteria used to judge whether, or how, to proceed with future definitive trial	5/6
Sample size	7a	Rationale for numbers in the pilot trial	
	7b	When applicable, explanation of any interim analyses and stopping guidelines	n/a
**Randomisation**			
Sequence generation	8a	Method used to generate the random allocation sequence	5
	8b	Type of randomisation(s); details of any restriction (such as blocking and block size)	5
Allocation concealment mechanism	9	Mechanism used to implement the random allocation sequence (such as sequentially numbered containers), describing any steps taken to conceal the sequence until interventions were assigned	5
Implementation	10	Who generated the random allocation sequence, who enrolled participants, and who assigned participants to interventions	5
Blinding	11a	If done, who was blinded after assignment to interventions (for example participants, care providers, those assessing outcomes) and how	n/a
	11b	If relevant, description of the similarity of interventions	n/a
Statistical methods	12	Methods used to address each pilot trial objective whether qualitative or quantitative	5
**Results**			
Participant flow (a diagram is strongly recommended)	13a	For each group, the numbers of participants who were approached and/or assessed for eligibility, randomly assigned, received intended treatment and were assessed for each objective	5
	13b	For each group, losses and exclusions after randomisation, together with reasons	5
Recruitment	14a	Dates defining the periods of recruitment and follow-up	5
	14b	Why the pilot trial ended or was stopped	n/a
Baseline data	15	A table showing baseline demographic and clinical characteristics for each group	5
Numbers analysed	16	For each objective, number of participants (denominator) included in each analysis. If relevant, these numbers should be by randomised group	7–8
Outcomes and estimation	17	For each objective, results including expressions of uncertainty (such as 95% confidence interval) for any estimates. If relevant, these results should be by randomised group	7–8
Ancillary analyses	18	Results of any other analyses performed that could be used to inform the future definitive trial	7–8
Harms	19	All important harms or unintended effects in each group (for specific guidance, see CONSORT for harms)	8
	19a	If relevant, other important unintended consequences	8
**Discussion**			
Limitations	20	Pilot trial limitations, addressing sources of potential bias and remaining uncertainty about feasibility	9–12
Generalisability	21	Generalisability (applicability) of pilot trial methods and findings to future definitive trial and other studies	9–12
Interpretation	22	Interpretation consistent with pilot trial objectives and findings, balancing potential benefits and harms and considering other relevant evidence	9–12
	22a	Implications for progression from pilot to future definitive trial, including any proposed amendments	9–12
**Other information**			
Registration	23	Registration number for pilot trial and name of trial registry	5
Protocol	24	Where the pilot trial protocol can be accessed, if available	5
Funding	25	Sources of funding and other support (such as supply of drugs), role of funders	2
	26	Ethical approval or approval by research review committee, confirmed with reference number	4

#### Quantitative methodology

Quantitative analysis of the recruitment phase was separated into an evaluation of screening logs and recruitment rates. Screening logs from each study site identified the processes involved in recruitment to the MATE trial. All study sites were requested to maintain a log of all participants approached to join the MATE trial and the outcome. An evaluation of the recruitment rates identified the processes surrounding total recruitment period, mean recruitment duration, recruitment rates per site and per month and enrolment risk time.

Success of the MATE pilot study was evaluated by achieving both of the following pre-specified formal progression criteria:

1. Recruitment of 80% of patients within the recruitment window (6 months)
2. A total of 20% or less withdrawal rate from the study at 2 years

To evaluate whether the MATE trial protocol and trial processes worked well for a future large-scale RCT, one of the following decisions was made based on the end of study data:

1. Stop — Main study not feasible
2. Proceed with modifications.
3. Proceed without modifications but with close monitoring.
4. Proceed without modifications.

A safety evaluation of the MATE pilot trial will report on the number of adverse and serious adverse events recorded for each treatment regimen.

#### Qualitative methodology

Qualitative analysis of the recruitment phase was separated into an evaluation of the feasibility of the recruitment and set-up phase and the running of the MATE trial. The PIs at each study site had overall responsibility for study activities, including recruitment. As such, only PIs were approached to complete the interviews rather than research nurses who assisted with running the study at each study site. Participation in this aspect of the study was on a voluntary basis with no adverse impact on funding or authorship if someone declined to take part. All trial staff who did take part provided written informed consent. All qualitative interviews, conducted by the main investigator and author AA, were in English, face to face and audio recorded and took place at the end of the recruitment phase, lasting between 5 and 22 min. A thematic analysis approach was used to analyse all interview transcripts [21] which were anonymised prior to labelling. All interviews were coded by two of the authors (A. A. and H. A. B.) until data-generated, key themes were agreed upon.

The secondary objective of the MATE trial was to report the outcomes of the two treatment arms (SC and T&E), evaluated by measurements of best-corrected visual acuity (BCVA) and central retinal thickness (CRT). Treatment burden was evaluated by the number of treatments and visits. These data are summarised using mean (SD) at each time point (baseline, 12 and 24 months) and the change from baseline to 12 and 24 months.

## Results: quantitative analysis

### Screening logs

A summary of the screening logs from each study site is shown in Table 2. The overall screen failure rate across all sites in the MATE trial was 18.5%, with the most common screen failure reason being visual acuity (VA) too good to qualify for the study (40%) followed by being unable to complete screening procedures (20%), specifically the (FFA) assessment.

**Fig. 3:**
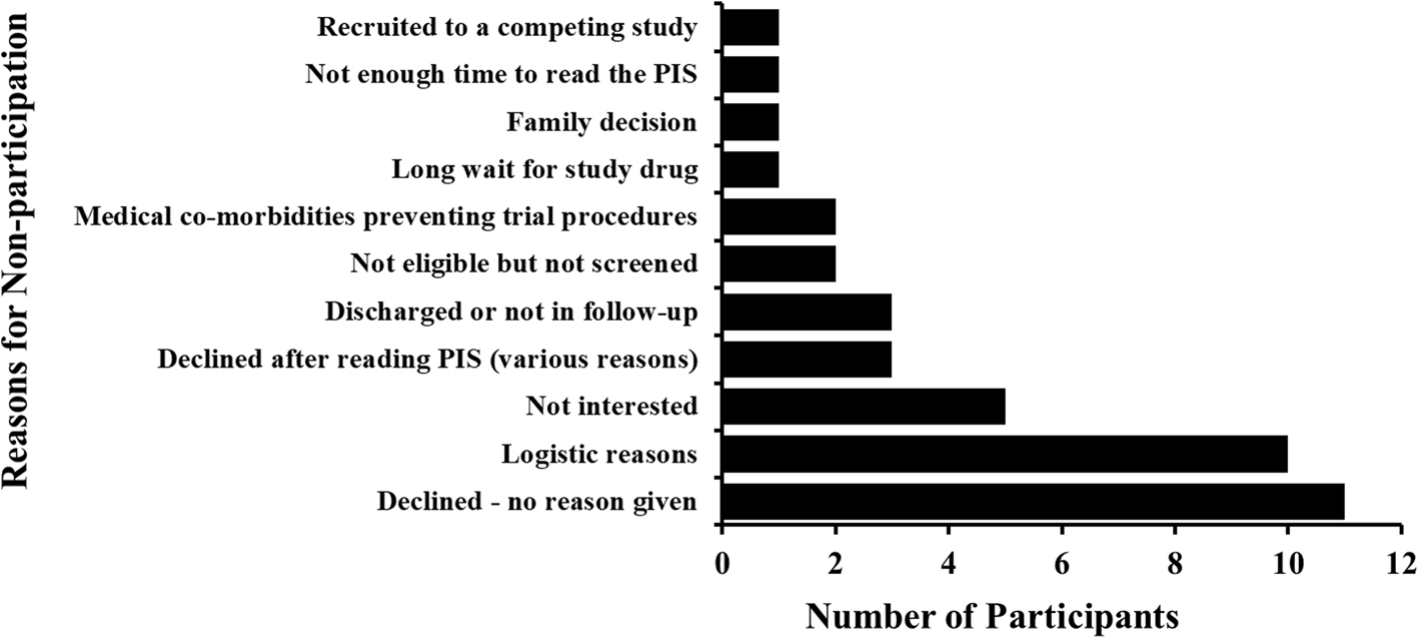
Bar chart summarising the reasons given for nonparticipation in the MATE trial

Non-participation rate was 40.8% (Fig. 3). In 28.9% of cases, no reason was given for not wanting to take part in the study. Logistical reasons accounted for 26.3% of cases, including travel-related concerns, screening appointment not at a convenient time and treatment preference in another hospital. A further 10% were not interested in the research with three participants declining to take part after reading the PIS due to concerns about risks, the length of the study and not wanting treatment.

### Recruitment rates

Mean recruitment duration was 194 days (*SD* = 100.8 days; range = 129–393 days; Table 2). The original recruitment window of 6 months was extended to 13 months to meet the recruitment target. During the final 4 months, all study sites competed against each other to recruit the remaining spaces available in the study. This meant that those sites who had already achieved their initial target were given the opportunity to over recruit to help meet the overall study target. Overall recruitment rates were approximately 3 participants per month with 6–7 participants recruited per study site.

In order to account for differing site activation dates, an enrolment risk time (ERT) was calculated as the time from site activation to overall study level enrolment cessation for each study site. This was divided by the number of participants enrolled at each site to calculate the enrolment risk per month (ERPM). The ERT varied from 4.6 to 13 months, with a mean of 6.45 months with the ERPM varying between 0.56 and 1.25 between study sites (Table 3).

**Table 3:** Summary of screening logs and recruitment details across all study sites involved in the MATE study

	**Study site**					
	**1**	**2**	**3**	**4**	**5**	**6**
**Screening log summary (total)**						
Offered PIS (93)	26	11	14	10	10	22
Declined (38)	13	3	5	2	4	11
Screened (54)	12	8	9	8	6	11
Screen fails (10)	1	0	1	3	1	4
Recruited (40)	11	8	8	5	3	5^a^
Withdrawals (4)	0	0	0	0	2^b^	3^c^
Other (1)	1^a^	0	0	0	0	0
**Recruitment summary**						
Site target	8	8	8	5	10	4
Site initiation	30 December 2015	06 May 2016	19 April 2016	06 May 2016	31 May 2016	31 May 2016
First screen	20 January 2016	08 June 2016	26 April 2016	24 May 2016	27 July 2016	05 July 2016
First consent	27 January 2016	08 June 2016	26 April 2016	02 August 2016	27 July 2016	05 July 2016
Last consent	25 January 2017	21 September 2016	08 November 2016	28 September 2016	06 October 2016	01 November 2016
Recruitment window	393	139	203	146	129	155
ERT (months)	13	4.63	6.76	4.86	4.30	5.16
ERPM (months)	1.18	0.56	0.87	1.25	0.70	1.00

*PIS* participant information sheet, *ERT* enrolment risk time, *ERPM* enrolment risk per month

^a^Offered PIS but recruited to competing study

^b^Withdrawn by study sponsor as non-refracted VA used at screening visit

^c^One participant after first visit, two participants by study sponsor as non-refracted VA used at screening visit

### Pilot RCT evaluation

The MATE trial achieved its recruitment target of 40 participants, albeit recruited over a more extended recruitment period than originally planned. Together, with a withdrawal rate of 15%, the MATE trial met both criteria for deeming it a success. However, to ensure a tighter recruitment window and facilitate the running of a future planned large-scale study, it was decided that a full-RCT version of the MATE trial could go ahead but with the following modifications:

1. Careful site selection with planned site selection visits to choose the appropriate teams and involvement of all stakeholders as early as possible in designing the study
2. Additional support from the sponsor team, favourable trial eligibility criteria and early monitoring systems to positively impact recruitment
3. Competitive recruitment between sites to boost recruitment
4. Sharing of good practice between sites in the form of newsletters, reminders for milestone visits, training and retraining of research teams to be up to date with trial specific procedures

### Safety evaluation

Across all six NHS sites involved in the MATE trial, a total of 225 adverse events (AEs) were recorded; 118 and 107 were recorded from the SC and T&E treatment regimens respectively (Table 4). A total of 39 serious adverse events (SEAs) were recorded across all study sites; 23 and 16 recorded from the SC and T&E treatment regimens respectively (Table 4).

**Table 4:** A breakdown of adverse and serious adverse events noted across all sites in the MATE trial and the number of patients affected. Patients with multiple adverse events in a particular category were only counted once in that category

	**SC treatment regimen**	**T&E treatment regimen**
**AEs (total no. of patients)**	16 (19)	18 (20)
Cardiac disorders	1	1
Ear & labyrinth disorders	1	2
Eye disorders	16	14
Gastrointestinal disorders	4	5
General disorders & administration site conditions	3	4
Hepatobiliary disorders	1	0
Infections & infestations	10	9
Injury, poisoning & procedural complications	7	4
Investigations	0	1
Musculoskeletal & connective tissue disorders	3	5
Neoplasms; benign, malignant & unspecified	1	0
Nervous system disorders	5	1
Reproductive system & breast disorders	0	1
Respiratory, thoracic & mediastinal disorders	3	2
Skin & subcutaneous tissue disorders	4	2
Surgical & medical procedures	3	5
Vascular disorders	1	2
**SAEs (total no. of patients)**	9 (19)	8 (20)
Cardiac disorders	3	1
Ear & labyrinth disorders	0	1
Eye disorders	1	0
Gastrointestinal disorders	1	1
Hepatobiliary disorders	2	0
Infections & infestations	3	2
Injury, poisoning & procedural complications	2	2
Neoplasms; benign, malignant & unspecified	2	2
Nervous system disorders	0	2
Renal & urinary disorders	1	1
Respiratory, thoracic & mediastinal disorders	0	2
Vascular disorders	1	0

*SC* standard care, *T&E* treat and extend, *AEs* adverse events, *SAEs* serious adverse event

## Results: qualitative analysis

To assess the feasibility of the recruitment and set-up phase, semi-structured interviews were conducted with seven key staff, including the trial manager, chief investigator (CI) who was also the principal investigator (PI) at the primary site and PIs at the remaining five study sites, resulting in seven interviews. To assess the feasibility of running the MATE trial, qualitative semi-structured interviews were conducted with key trial staff including the study sponsor team comprising of the trial manager, study monitor and sponsor representative, the CI who is also the PI at the primary site along with each PI at the remaining study sites. The resulting nine interviews were analysed alongside an additional three interviews collected at the end of the recruitment phase from the lead pharmacy representative, trial manager and CI.

### Recruitment and set-up phase

A thematic analysis from the seven interviews established four key themes relating to the recruitment and set-up phase of the MATE trial. The key themes identified were recruitment processes, protocol-related factors, human factors and challenges, with individual items related to each key theme listed in Table 5. These outcomes highlight that an individualised recruitment strategy tailored to each study site and specific to the study is essential in ensuring a successful recruitment strategy. Recruitment is also facilitated by minimising delays, training trial personnel about study procedures, good communication between study sponsor and teams and favourable study design features.

**Table 5:** Key themes and individual themes relating to the recruitment and set-up phase and the running of the MATE study identified from semi-structured interviews with key members of the study team

	**Main themes**	**Sub-themes**
**Recruitment and set-up phase**	Recruitment processes	Recruitment target
		Recruitment strategy
		Ease of recruitment
		Recruitment period
	Protocol-related factors	• Eligibility criteria
		• Standard NHS treatment and licensed treatment
		• Protocol breach
		• Randomisation and processes around randomisation
		• Patient perspective on randomisation
	Human factors	• Investigator bias
		• Communication between sites and sponsor
		• Dedicated members of research teams recruiting patients
	Challenges	• Delays
		• Limited resources
		• Site withdrawals
		• Patient withdrawals
		• Competing for PIs time
		• Building teams
**Running the MATE study**	Variation	• Individual site setup and local NHS AMD treatment service delivery pattern
		• Variation in practice at sites in comparison with MATE study and between sites
		• Support to teams and level of engagement with sponsor
		• Documentation — GCP validity and recording of AEs
		• Individual site research experience and level of research activity
		• Challenges due to variation — protocol deviation and capacity
	Challenges	• Staff turnover — both PI and nursing
		• Protocol-related deviations and data quality
		• Limited resources — staff and finances
		• Clinical trial planning — continuity of care

*PI* principal investigator, *AMD* age-related macular degeneration, *GCP* good clinical practice, *AEs* adverse events

### Running the MATE study

A thematic analysis of the twelve interviews identified two key themes related to running the MATE trial. These key themes were variation and challenges, with individual items relating to each key theme outlined in Table 5. Variation in research delivery, site set-up and research team composition can affect delivery of a clinical trial. Liaising with study teams early in the clinical trial journey to understand their research team and resources allows for modifications to the study protocol where possible to fit their needs.

## Results: secondary objective

### BCVA

**Fig. 4:**
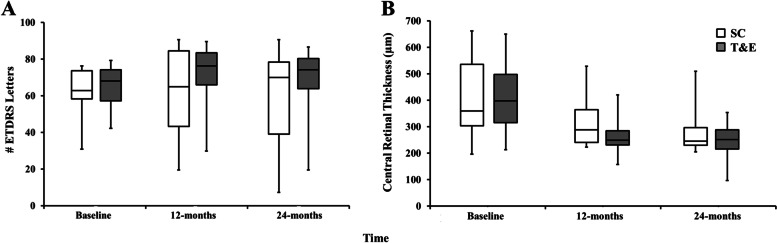
Box plot showing the best-corrected visual acuity measured in the number of ETDRS letters (**A**) and central retinal thickness (**B**) measured over time. White boxes represent data from the standard care (SC) treatment regimen with grey boxes representing data from the treat-and-extend (T&E) treatment regimen. For all box plots, the horizontal black line denotes the median value with the 25th and 7th percentiles. Error bars represent the minimum and maximum values

In the SC group, mean BCVA was 60.8 (*SD* = 12.5) ETDRS letters at baseline, 60.8 (*SD* = 21.3) at 12 months and 58.0 (*SD* = 25.4) at 24 months. The mean change in BCVA from baseline was + 0.7 (*SD* = 18.6) and − 2.4 (*SD* = 23.6) ETDRS letters at 12 and 24 months, respectively (Fig. 4A). Compared to baseline, a gain of 15 ETDRS letters or more was found in 3 out of 17 eyes (18%), with 5 out of 17 (29%) eyes losing 15 letters or more. In eyes losing 15 letters or more, the reasons were neovascular reactivation (2 × patients) with one each also having fibrosis, atrophy and retinal pigment epithelial (RPE) rip.

In the T&E group, mean BCVA at baseline was 63.7 (*SD* = 10.0) ETDRS letters, increasing to 69.3 (*SD* = 15.8) letters at 12 months and reducing slightly to 65.8 (*SD* = 18.3) letters at 24 months. The mean change in BCVA from baseline was + 5.7 (*SD* = 15.6) letters at 12 months and + 2.9 (*SD* = 19.2) letters at 24 months (Fig. 4A). Compared to baseline BCVA, a gain of 15 ETDRS letters or more was found in 5 out of 18 eyes (28%), with 3 out of 18 (17%) eyes losing 15 letters or more. In eyes losing 15 letters or more, the reasons were fibrosis and macular haemorrhage (2 × patients).

## CRT

In the SC group, mean CRT at baseline was 414.3 µm (*SD* = 144.5 µm) decreasing to 308.9 µm (*SD* = 83.5 µm) at 12 months and 277.6 µm (*SD* = 78.4 µm) at 24 months. The mean change in CRT from baseline was − 116.5 µm (SD = 111.2 µm) and − 148.8 µm (SD = 122.5 µm) at 12 and 24 months, respectively (Fig. 4B).

In the T&E group, mean CRT at baseline was 406.6 µm (*SD* = 114.6 µm) decreasing to 258.8 µm (*SD* = 52.5 µm) at 12 months and 247.6 µm (*SD* = 56.7 µm) at 24 months. The mean change in CRT from baseline was − 147.8 µm (*SD* = 104 µm) at 12 months and − 164.8 µm (*SD* = 117.8 µm) at 24 months (Fig. 4B).

### Treatment burden

The mean number of treatments and visits was 8.3 (*SD* = 0.7) and 9.5 (*SD* = 1.8), respectively, at 12 months and 17.3 (*SD* = 2) and 16.4 (*SD* = 3.8), respectively, at 24 months, for the SC and T&E groups, respectively.

## Discussion

The MATE trial was designed as a pilot, RCT employing a mixed methodology to evaluate the requirements to inform a large-scale RCT comparing standard care (SC) with a treat-and-extend (T&E) regimen of aflibercept for nAMD. Qualitative and quantitative analyses revealed the MATE trial achieved pre-defined criteria deeming the pilot study a success. Following some minor recommendations, the MATE trial protocol can progress to a large-scale RCT.

Maintaining screening logs has been recommended as good practice [7, 22] providing information relating to screen fails and non-participation rates. Screen fail rates in the MATE trial were 18.51%, lower than similar previous studies [23–29]. The most common cause of screen fails was not meeting the eligibility criteria, in line with previous research [27, 29, 30]. Protocol amendments aimed to facilitate recruitment are most often made regarding eligibility criteria and account for 16% of all protocol amendments [31]. To facilitate recruitment in the MATE trial, three protocol amendments were made. Firstly, the screening and baseline visit were amended to take place on the same day, reducing participant burden with the need for an additional study baseline appointment. Secondly, eligibility blood pressure (BP) criteria were deemed too strict, thus impeding recruitment. As such, BP criteria were reduced from > 160 mmHG to > 170 mmHG to facilitate recruitment. Finally, the recruitment window was extended from the original 6 months to 13 months to enable participating study sites to reach their recruitment target (see Supplementary Table 1). Screen failures can have financial implications both for the sponsor and the study site [31] including not being paid for participants who fail screening despite the work being carried out. Consideration should therefore be made during the study planning stage if there is a high possibility of screen failures for the study as sites may require an agreement to assist with the additional workload.

Nonparticipation is another important measure acquired through screening logs, providing insight into why participants decide not to take part in a study. Non-participation rate in the MATE trial was 40.8% which is far lower than previous studies reported [24–26, 28]. The most common reason for nonparticipation was the patient declining to take part, accounting for 92%. In contrast to previous studies [28, 32], there were no instances of investigators declining to recruit to the MATE trial. The second most common reason for nonparticipation is related to logistics, for example travel and inconvenient appointments. Recording these reasons via screening logs resulted in a protocol amendment early in the MATE trial to facilitate recruitment, enabling screening and baseline visits to take place on the same day where possible to minimise patient and staff burden.

Recruitment rates are useful in the planning of future RCTs to know the target and time required to recruit to target for similar nAMD treatment trials. Recruitment rates indicate that 3.07 participants were recruited per month in the MATE trial. Whilst this rate was lower compared to previous studies, the recruitment rate per study site per month was in fact better [19, 33, 34]. This difference in monthly recruitment rates may be explained by the fact that all the previous studies were multinational trials involving a large number of study sites. As a result, more patients were recruited per month overall even though individual study sites recruited fewer patients per month. Regarding the recruitment period, the original duration in the MATE trial was 6 months, but this was extended to 13 months in order to meet the target recruitment of 40 participants. We also established that once a study site becomes active for recruitment, priority should be given to recruit the desired number of participants within the minimum time possible; this is useful advice for future RCTs.

The recruitment process is a complex interplay of human factors, regulatory factors and study design. Reducing delays, training trial personnel about study procedures, good communication between the sponsor and research teams and favourable study design features all facilitate recruitment. The qualitative aspect of the MATE trial highlights the need for an individualised recruitment strategy tailored specifically to each study site for a given study. Our results also suggest that recruitment was positively impacted by investigator bias and inability to convey equipoise between the two regimens to trial participants by the recruitment team. The MATE trial allowed participants to begin treatment despite VA levels being better than that recommended by NICE guidelines, which at the time were 6/96 and 6/12. During our study, some PIs also preferred the T&E regimen in everyday practice and were more likely to recruit eligible patients to the MATE trial. Whilst this experience has similarly been reported in research into other specialities, they state that investigator bias and inability to convey equipoise had a negative impact on study recruitment [35–37]. Mitigating these factors in future RCTs can be achieved by providing study specific training on informed consent to the trial staff involved in recruitment processes and additional information aids for potential participants.

Delivering a clinical trial can be affected by a number of variables including variation in research delivery, site set-up and research team composition. Liaising with study teams early in the clinical trial journey is helpful, particularly in the protocol development stage to understand each research team and its resources in order to modify the study protocol where possible to fit their needs. Our qualitative interviews also highlighted the varied level of experience in conducting clinical trials amongst the participating study sites. This resulted in some sites requiring more support and training throughout the study to ensure commitment remained stable. This was also evident by the sites with previous clinical trial experience recruiting with fewer hurdles. Choosing sites carefully is therefore one of the recommendations for taking the MATE pilot to a full-scale RCT. With busy departments like ophthalmology, resources are stretched, and factoring in the variation in practice at the different sites is useful to ensure smooth delivery of a clinical trial and support the teams better. Staff turnover at sites is a challenge in trials running for longer durations. For example, in the MATE trial, we faced a high research nurse turnover at one study site that changed their research nurse four times in the period of one year. The role of research nurses in the MATE trial was to assist the site PIs in running the study, checking vital signs and facilitating the clinical visit. Therefore, our clinical trial manager supported this team in the form of regular training of new members of staff about the trial-specific procedures and with regular phone calls and reminder emails of study milestones. Having a data management plan early in the study and the flexibility to monitor any sites more often if there were issues with data quality also help in protocol adherence and supporting the teams.

The secondary objective of the MATE trial compared BCVA, CRT, treatment burden and the number of visits between the two treatment arms. The SC regimen showed a mean visual gain of + 0.7 ETDRS letters at 12 months, with a decline of − 2.4 letters at 24 months. This is not in keeping with other studies evaluating a similar regimen, such as the 2q8 arm of the VIEW study and real-world data, which reported a mean gain in visual acuity [18, 38, 39]. This can be explained by outliers in the SC regimen of the MATE trial: five patients lost more than 30 letters from baseline; of these, two patients had a reactivation of the neovascular activity in the second year, and of the other three, one patient had fibrosis, one had atrophy and another had a RPE rip. Fewer treatments in real-world studies may reflect the variability between clinicians and centres in implementing a T&E regimen in the second year [38, 39], yet aggressive treatment in the second year maintains the visual acuity gains achieved in the first year [39].

The T&E regimen showed a mean visual gain of + 5.7 ETDRS letters at 12 months and + 3 letters at 24 months, achieved with a mean of 9.5 treatments in the first year. This visual gain is in keeping with other studies evaluating a T&E regimen, such as the ALTAIR study [40]. However, the 2-weekly extension arm showed a gain of 9 letters at 52 weeks with a mean of 7.2 treatments. The ATLAS [41] study, a prospective, multicentre, open-labelled study evaluating a T&E regimen of aflibercept, showed similar visual gains at year 2. The ability to extend treatment intervals to 12 weeks is consistent with other prospective studies with a similar regimen [40, 41]. Barthemes et al. and Mekjaic et al. also demonstrate a mean visual gain with aflibercept using T&E regimen with 13.6 and 14.5 treatments in 2 years [42, 43]. Barthelmes et al. were able to extend approximately one-fourth of the cohort to a treatment of 12 weeks or more [42].

Lessons learned from conducting the MATE pilot trial have led to the following 6 recommendations:

1. During the study setup stage, careful site selection with planned site selection visits helps in choosing the right teams and getting a firmer commitment from sites.
2. Involving all stakeholders at an early stage, where possible, from a protocol development stage is useful in considering variations in local care delivery.
3. Planning regulatory approvals and opening new sites to maintain a tighter and shorter recruitment window, for example timing the opening of a site to fit with investigator annual leave or competing studies at a site
4. At the recruitment stage, good support from the sponsor team, favourable trial eligibility criteria (for example visual acuity entry criteria better than NICE guidance in this study) and having early monitoring systems in place all have a positive impact on recruitment. Another strategy to boost recruitment found to be useful in our study was opening up the study for competitive recruitment as sites are keen on meeting their individual recruitment target.
5. Sharing of good practice between sites in the form of newsletters, reminders for milestone visits, training and re-training of research teams to be up to date with trial-specific procedures are helpful in smooth delivery of a study.
6. Adapting the amount and nature of sponsor support to the individual site needs is recommended during the study.

To conclude, employing a mixed methodology in the MATE pilot trial has provided novel and valuable insight into the recruitment phase and conduct of running a RCT. We find that the current study protocol will be deliverable with some minor changes as outlined above in the recruitment and running of the future planned large-scale RCT study to efficiently compare two treatment arms of aflibercept for nAMD.

## Supplementary Information

**Additional file 1:Supplementary Table 1.** A full list of inclusion and exclusion criteria for the MATE trial. **Supplementary Table 2. **List of protocol amendments which impacted recruitment to the MATE trial.

## Data Availability

The datasets analysed during the current study are available from the corresponding author on reasonable request.
