# Supplementary material for: The MATE trial: a multicentre, mixed-methodology, pilot, randomised controlled trial in neovascular age-related macular degeneration
Source: Pilot Feasibility Stud. 2023 Apr 20;9:63. doi: 10.1186/s40814-023-01288-0 (PMC10116669; doi:10.1186/s40814-023-01288-0)
Supplement: Supplementary file 1 — Additional file 1:Supplementary Table 1. A full list of inclusion and exclusion criteria for the MATE trial. Supplementary Table 2. List of protocol amendments which impacted recruitment to the MATE trial. [file 40814_2023_1288_MOESM1_ESM.docx]

**Supplementary Table 1:** A full list of inclusion and exclusion criteria for the MATE trial.

| **Inclusion Criteria** |
| --- |
| - Visual Impairment predominantly due to nAMD |
| - Active, treatment naïve, angiographically active choroidal neovascular membrane in the study eye secondary to nAMD with any part of the lesion or its sequelae (e.g. SRF, haemorrhage, PED, sub-RPE fluid) in a sub-foveal location |
| - Visual acuity of 78-24 ETDRS letters at screening and baseline in the study eye |
| - Age ≥ 50 years |
| - Able to provide written informed consent to the study |
| - Able and willing to attend for hospital visits at the frequency required |
| - If both eyes are eligible at baseline, the eye with worse visual acuity will be the study eye, although the final decision will rest with the investigator. Any deviation from entering the eye with worse visual acuity at baseline into the study will be explained and documented in the patient notes and the case report forms (source data). The choice of eye selected for inclusion into the study will be determined and documented before the patient is randomised. A patient who has both eyes that may be eligible may therefore undergo a different treatment regimen in eye; however, they will be treated with aflibercept in both eyes. Hospital visits will be co-ordinated to minimise the number of attendances required and therefore the inconvenience for the patient. |
| **Exclusion Criteria** |
| - Inability to comply with the study or follow-up procedures |
| - Pregnant or lactating women |
| - Women of childbearing potential, unless they are using effective methods contraception during treatment and for 90 days after their last injection (Effective methods include male sterilization, female sterilization, intrauterine device, oral, injectable or implantable hormonal methods of contraception where inhibition of ovulation is the primary mode of action), total abstinence (only if it is the patient’s preferred and usual lifestyle, i.e. not a declaration of abstinence for the duration of the trial, periodic abstinence or withdrawal) |
| - Males with female partners of childbearing potential who do not agree to an effective form of contraception during treatment and for 90 days after their last injection |
| - Previous treatment for CNV in the study eye |
| - Fibrosis consisting of more than 50% of the lesion or involving the centre of the fovea |
| - Co-existing pathology within 0.5 disc diameters of the fovea that could prevent an improvement in visual acuity in the opinion of the investigator (e.g. macular hole, dense epiretinal membrane) |
| - Cataract (causing significant visual impairment), aphakia, vitreous haemorrhage, reitnla detachment, proliferative retinopathy or CNV due to any cause other than AMD at screening and baseline |
| - Known allergy to aflibercept or fluorescein |
| - History of cerebrovascular accident, transient ischemic attack or myocardial infarction within 3 months of the screening visit |
| - Any type of systemic disease or treatment that may affect or expect to affect the clinical status of the patient to a significant degree |
| - Blood pressure of >170mmHg systolic or >110mmHg diastolic at screening or baseline |
| - Any active periocular infection or inflammation at screening or baseline |
| - Uncontrolled glaucoma (30mmHg) at screening or baseline |
| - Neovascularisation of the iris at screening or baseline |
| - Treatment with any anti-angiogenic drugs to either eye within 3 months of baseline |
| - Nd-YAG laser capsulatomy within the last 2 months or expected within 6 months of baseline in the affected eye |
| - Use of other investigational drugs within 30 days |
| - Use of systemic anti-VEGF agents within 3 months prior to baseline |
| - Use of systemic corticosteroids for at least 30 consecutive days within the 3 months prior to baseline |
| - Current or planned medications known to be toxic to the lens, retina or optic nerve, e.g. hydroxychloroquine, desferoxamine, tamoxifen or ethambutol |

**nAMD: Neovascular age-related macular degeneration; SRF: Subretinal Fluid; PED: Pigment Epithelial Detachment; RPE: Retinal Pigment Epithelial; ETDRS: Early Treatment Diabetic Retinopathy Study; CNV: Choroidal Neovascularisation; AMD: Age-related Macular Degeneration; VEGF: Vascular Endothelial Growth Factor*

**Supplementary Table 2:** List of protocol amendments which impacted recruitment to the MATE trial.

| **Amendment Description** | **Benefit to Recruitment** |
| --- | --- |
| Screening and baseline on the same day | This benefited recruitment and reduced burden on patients by eligible participants being able to complete screening and baseline procedures on the same day rather than arranging additional appointments. |
| Blood pressure (BP) changes from systolic >160mmHg to >170mmHg | Stricter BP criteria were thought to impede recruitment to the trial. The amendment to reduce the BP during the latter half of the recruitment period facilitated recruitment. |
| Extension of recruitment window to 28 Feb 2017 | This extended the recruitment window from the original 6 months to 13 months. This gave all participating sites more time to hit their own target whilst a competitive recruitment strategy between sites enabled the MATE trial to hit its overall recruitment target. |
